# The New Frontiers in Neurodegenerative Diseases Treatment: Liposomal-Based Strategies

**DOI:** 10.3389/fbioe.2020.566767

**Published:** 2020-10-26

**Authors:** Mariafrancesca Cascione, Valeria De Matteis, Stefano Leporatti, Rosaria Rinaldi

**Affiliations:** ^1^Department of Mathematics and Physics “Ennio De Giorgi,” University of Salento, Lecce, Italy; ^2^National Research Council Nanotec Institute of Nanotechnology, Lecce, Italy

**Keywords:** neurodegenarative diseases, Blood Brain Barrier (BBB), liposome, solid lipid nanoparticles, nanostrured lipid particles, ethosomes, drug delivery

## Abstract

In the last decade, the onset of neurodegenerative (ND) diseases is strongly widespread due to the age increase of the world population. Despite the intensive investigations boosted by the scientific community, an efficacious therapy has not been outlined yet. The drugs commonly used are only able to relieve symptom severity; following their oral or intravenous administration routes, their effectiveness is strictly limited due to their low ability to reach the Central Nervous System (CNS) overcoming the Blood Brain Barrier (BBB). Starting from these assumptions, the engineered-nanocarriers, such as lipid-nanocarriers, are suitable agents to enhance the delivery of drugs into the CNS due to their high solubility, bioavailability, and stability. Liposomal delivery systems are considered to be the ideal carriers, not only for conventional drugs but also for neuroprotective small molecules and green-extracted compounds. In the current work, the LP-based drug delivery improvements in *in vivo* applications against ND disorders were carefully assessed.

## Introduction

Pharmacological research is oriented to develop new effective therapeutic strategies in order to treat diseases and, at same time, to improve the life quality of patients. Many studies aim to discover new drugs, taking in consideration that their effectiveness is strictly dependent on the administration route and also on their intrinsic ability to access the organs and tissues in suitable amounts and times. The uptake of drugs is particularly difficult in specific districts of body, such as the Central Nervous System (CNS), in which the presence of a Blood Brain Barrier (BBB) constitutes the major obstacle. The BBB often inhibits neuroreparative and neuroprotective therapies that should carry out their pharmacological action directly on target site. According to the latest available data, it is estimated that 10 % of the world population will be affected by neurological disorders, without geographical and socioeconomic distinctions (Organization, 2006). For this reason, the World Health Organization recommends great efforts to overcome the difficulties in the brain drugs administration. Despite the neuropharmaceutical field constituting the largest sector of drug industry, its development is limited by the complication regarding the BBB crossing. It has been estimated that ca. 98% of the drugs available today for the treatment of neurological diseases, including recombinant proteins, monoclonal antibodies, and genes are not able to effectively overcome the BBB. This is true due to their deficiency to exploit specific transport mechanisms and their high molecular weight together with polarity (Pardridge and Boado, 2012). In light of this, the regulatory mechanisms of the BBB are fundamental for the development of new therapies dedicated to large groups of brain injuries such as neurodegenerative diseases.

## Neurodegenerative Diseases

The age-dependent disorders are becoming increasingly prevalent, in part because the elderly population has increased in recent years (Heemels, 2016).

Examples of neurodegenerative diseases are amyotrophic lateral sclerosis, Parkinson's disease, Alzheimer's disease, Huntington's disease, frontotemporal dementia, and the spinocerebellar ataxias. All these pathologies are different in pathophysiologic properties but, at the same time, are associated with memory loss, cognitive impairments, and other adverse effects including a person's inability to move, breathe, and speak (Abeliovich and Gitler, 2016; Wyss-Coray, 2016; Gitler et al., 2017). These pathologies show selective neuronal vulnerability combined with the degeneration of specific brain regions; in addition, an abnormal protein deposit (extracellular or intracellular) occurs in neurons or other types of brain cells (Ross and Poirier, 2004).

Alzheimer's disease (AD) is characterized by neuron degeneration; in particular, in the basal forebrain and hippocampus. In addition, the alteration of synaptic and neuronal connections is involved in the pathogenesis (Selkoe, 2002). The accumulation of amyloid-β (Aβ) peptides and tau protein boosts the development of the pathology progression (Combs et al., 2016). In detail, the Amyloid beta, a 39–43-amino acid residue peptide, is a physiological presence in human brain and is derived from the proteolysis process of the amyloid precursor protein (APP). The extracellular accumulation of amyloid-β (Aβ) peptides induces the formation of amyloid plaques, also called senile plaques, which form the synaptic dysfunction and neuronal loss (Citron, 2002). The tau proteins (T proteins) play a key role in microtubule stabilization and they are abundant in hippocampal areas, cortical areas, and the entorhinal cortex. In AD disease, this protein undergoes a hyperphosphorylation phenomenon that induces tau protein clumping with the consequence of developing intracellular neurofibrillary tangles (NFT): these formations lead to axon degeneration and microtubule disaggregation (Goedert, 2004). The onset of AD may be due to rare genetic mutations, which were found in the APP and in some transmembrane proteins (presenilins) that cleave the APP. Mutations of tau28 protein have been found in non-Parkinson frontotemporal dementia.

Parkinson's disease (PD) is provoked by degeneration of dopaminergic neurons in the substantia nigra of the midbrain and other monoaminergic neurons in the brain stem (Vernier et al., 2004). Despite several progresses, the main cause of PD remains unknown. It is probably born by complicate connections between environmental and genetic factors that are strong effects on cells that trigger mitochondrial dysfunction, oxidative stress, impairment of the ubiquitin proteosome process, as well as defective autophagy process (Dawson et al., 2010; Angeles et al., 2014). However, the exact mechanisms leading to neuronal death are unknown. As a consequence, the loss of several neurotransmitters and neuromodulators in the extranigral neurons, such as noradrenaline (NA), acetylcholine (ACh), glutamate, and serotonin (5-hydroxytryptaminc [5-HT]) occur. These phenomena induce the slowing of movement, muscular rigidity, and tremor (Alexander, 2004).

Numerous gene mutations could induce the early onset of PD, in particular, point mutations or increased gene dosage of the α-synuclein gene. These boost an autosomal dominant PD. Instead, the mutations of genes encoding for DJ-1 or PINK132 and parkin could trigger a recessive early-onset PD. In general, the adult PD onset regards an inclusion body near the nucleus of neurons, immersed in cytoplasm, called Lewy body, in the form of fibrillar aggregates. Several investigations demonstrated the presence of Lewy bodies in the substantial nigra in monoaminergic, cerebral, cortical, and other neurons principally constituted of α-synuclein protein (Stefanis, 2012).

## The Blood Brain Barrier (BBB)

The human brain contains about 100 billion of capillaries with a total length of 600 km. This dense vascular network covers an area of about 20 m^2^ and represents an effective interface between the blood and the brain, supplying brain cells with oxygen and essential metabolites that support brain functions (Risau and Wolburg, 1990). In addition, the brain consumes 20% of the body's total glucose, increasing the blood flow and oxygen in order to quickly reach the different body's districts (Begley and Brightman, 2003). Capillaries are the major site of blood barrier brain (BBB) that constitutes a real barrier between the bloodstream and the central nervous system acting as selective filter, allowing or preventing substances (ions, glucose, proteins etc.) to move from the blood to the brain parenchyma and to cerebrospinal fluid (CSF). Thanks to this selectivity, the BBB protects the sickly chemical–physical homeostasis of the cerebral fluid environment carrying out a protective role toward CSF and the nervous tissue (Abbott and Romero, 1996).

This can be satisfied by keeping the ionic environment stable and preserving the low amino-acid gradient of the excitatory neurotransmitters (glutamic acids, aspartic acid, and glycine) that is characteristic of the brain extracellular fluid. This phenomenon is critical for reliable synaptic transmission and efficient neuroregulatory activity. BBB prevents the toxic compound uptake in cells, such as metabolites and neurotoxins both endogenous and xenobiotic, which are potentially fatal. In addition, the BBB promotes the longevity of the CNS and prevents premature cell death and neurodegeneration.

The BBB is formed by the endothelium of the brain capillaries, the processes perivascular of the astrocytes surrounding the endothelial cells and from the pericytes which are contractile connective cells that partially surround them. The anatomical structure of the BBB is responsible for its functional peculiarities, such as limited permeability to most substances and limited paracellular and transcellular transport (Zlokovic, 2008; Kisler et al., 2017). The brain capillaries are anatomically different from peripheral systemic ones since the cells that compose them form a continuous non-fenestrated endothelium, with a reduced number of pinocytic cells (Engelhardt and Liebner, 2014). Brain endothelial cells are connected by tight and adherent junctions. The first prevents the free diffusion of solutes from the blood sector (peripheral or systemic) to the CSF and brain (intrathecal), both at the level of the cerebral capillaries and the chorioid epithelium. The tight junctions involve occluding, claudin I, claudin 3, claudin 5, claudin 12, and the membrane-associated guanylate kinases, proteins ZO1, ZO2, and ZO3. The adherent junctions include cadherins, platelet endothelial cell adhesion molecules (PECAM1), and the junctional adhesions molecules (JAMs) JAMA, JAMB, and JAMC. The gas exchange (oxygen and carbon dioxide) occurs across the endothelium, whereas the pinocytosis deficit is responsible for the limitation of solutes passage (Abbott et al., 2010; Zhao et al., 2015).

Then, in order to target a specific drug to the brain, it is necessary to consider the structural and functional characteristics of the BBB and, at the same time, evaluate the chemical-physical properties of the drug (pKa, molecular weight, lipophilicity, etc.). In addition, it is important to consider the intrinsic ability of BBB to form bonds with the plasma proteins that prevent the CNS crossing, the degree of ionization (pH), and the lipid/water partition coefficient of the drug (Warren, 2018). In particular, it has been demonstrated that small lipid-soluble molecules (high partition coefficient) with a molecular weight <400 Da or containing <8 hydrogen bonds can cross BBB by simple transmembrane diffusion. Contrary, the drugs with low passive diffusion coefficient can be internalized in the brain only by active transports systems (Pardridge, 2015).

## Transport Through the BBB

**Figure 1:**
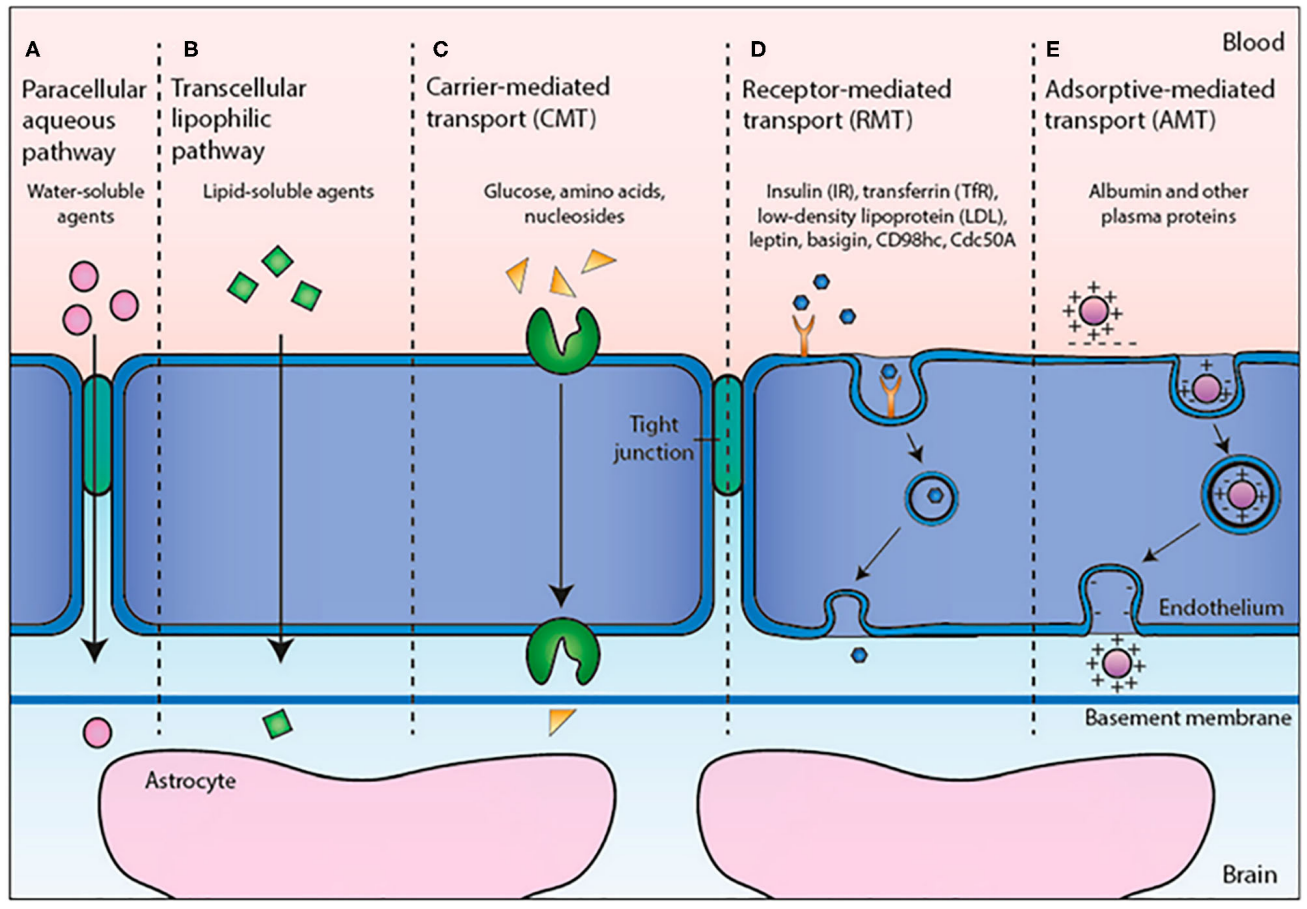
Transport mechanisms through BBB. Reproduced with permission from Cavaco et al. (2020).

Oxygen, carbon dioxide, glucose, nucleosides, vitamins, and some of fat-soluble drugs can cross the BBB by passive diffusion mechanisms or by specific transport mechanisms. The transport systems (Figure 1) can be located on the luminal or abluminal side of the BBB and can be classified into three categories (Abbott and Romero, 1996; Begley, 2004): (i) CMT (Carrier Mediated Transport) consists of a transport mediated by specific transport carrier proteins; they are able to move carbohydrates, fatty acids, amino acids, nucleotides, hormones, vitamins, organic cations and anions through BBB; (ii) AET (Active Efflux Transport) consists of an active transport mechanism capable of expelling a large variety of molecules from the brain compartment to the bloodstream; and (iii) RMT (Receptor Mediated Transport) which consists of receptors mediated transport system, able to drive large compounds (peptides and proteins) through an intracellular process in both directions: insulin and transferrin from blood to brain, as well as apolipoproteins (Demeule et al., 2008; Sagare et al., 2012). The transport of native plasma proteins or peptides is limited, but the cationization phenomenon can increase their uptake by adsorptive-mediated transport (AMT).

The CMT and AET systems are responsible for small molecule transport between the blood and the brain, while RMT allows large molecule delivery through the BBB. Among these, the transmembrane transporters called ABC transporters, expressed at the luminal side of BBB, play an important role due to the presence of two cytoplasmic domains useful to bind the ATP boosting transport against the unidirectional gradient (from cytoplasm to extracellular space) (Abbott et al., 2010; Mokgokong et al., 2014).

Therefore, ABC prevents the brain accumulation of xenobiotic agents, and drugs exert an important role in the body detoxification. The first ABC transporter identified is the P-glycoprotein (P-gp or ABCB1) coded by the MDR1 gene. This gene is known for its several polymorphisms (ca. 50) at the level of a single nucleotide, and for this reason, it is responsible for a strong individual variability in absorption and tolerance to drugs (Yan-Hong et al., 2006; Bartels, 2011). In addition to the ABC, OATP (Organic Anion Transporting-Polypeptide) and the OAT (Organic Anion Transporter) members are expressed in the endothelial cells of the BBB. The main function of OATP 1A2 and 2B1 carriers is to uptake several endogenous and xenobiotic compounds (10.1021/acs.molpharmaceut.0c00159), but, unlike the members of the ABC family, the OATP does not hydrolyze ATP, and consequently, they fail to uptake drugs against the concentration gradient (de Boer et al., 2003). Their presence allows the ions exchange according to the ionic gradient. In addition to the possibilities of exploiting or inhibiting these and other physiologically present transport mechanisms on the BBB (Pardridge and Boado, 2012), other approaches that can guarantee are being studied for a more effective delivery of drugs to the CNS. A spread class of carriers, abundant in mammalian neurocytes and brain capillary epithelium membrane, is also represented by glucose transporter proteins (GLUTs), GLUT1, and GLUT3 (McEwen and Reagan, 2004).

The approaches to achieve effective CNS drug concentrations can be invasive (temporary break of tight junctions, intracerebral injection, or use of intracerebral implants such as catheters, microchips, or erodible polymeric systems). It still comes cost-effective and potentially dangerous for patients, since the direct delivery of the drug exposes patients to the risk of developing serious brain infections with a consequent decrease in their compliance. Starting from these assumptions, the non-invasive (chemical, biological, or technological) approach is the hope to treat the brain pathologies (Scherrmann, 2002; Tosi et al., 2011).

In the last years, the use of nanoparticles (NPs) represents a powerful approach to improve the BBB penetration of bioactive molecules; NPs have small size (3 ÷ 200 nm) and good stability, and they can be easy functionalized by surface modifications (attaching active ligands or changing the superficial charge), offering good advantages over the free drug. The NPs can reduce the side effects on healthy cells, improving the pharmacokinetic and pharmacodynamics drug profile and increase drug concentration at the specific sites allowing their application in the neurodegenerative disease treatment.

## Lipid Nanocarriers for Drug Delivery Across the BBB

The effectiveness of drugs acting on the CNS has strong limits, due to their inability to overcome the BBB in adequate amounts (de Boer and Gaillard, 2007). The issue of the limited drugs crossing through the BBB is of great interest to the scientific and medical community, which is focused on the execution and optimization of innovative therapeutic strategies against neurodegenerative diseases. In this perspective, the carriers with dimensions at nanoscale level represent a promising drug delivery platform solution for enhancing the drug crossing through the BBB (Chen and Liu, 2012; Pardridge, 2012; Upadhyay, 2014). Theoretically, the tuneable physicochemical properties of nanocarriers permit optimizing the delivery of different kinds of drugs at the specific therapeutic target; nevertheless, a careful design of drug nanocarriers needs to consider many factors, such as biocompatibility, reduction of drug toxic effects, enhancement in drug stability, and optimization of the specific target functionalization. Furthermore, the engineering of such nanocarriers become harder when the therapeutic target is the CNS, as listed by Lockman and colleagues (Lockman et al., 2002).

The growing interest in the suitable drug delivery systems research has encouraged the production of a wide range of nano-scaled carriers (Mishra et al., 2010). Among these, lipid-based carriers are recognized as promising strategies for drug delivery, due to their ability to mimic natural lipid environment constituting the biomembranes, including BBB (Pujara, 2012). In addition, lipid vectors protect the drug from degradation during transport to the target sites, reduce the toxicity, and increase the drug biocompatibility with respect to free administration (Chen et al., 2010; Nunes et al., 2017).

Among different lipid carriers, liposomes (LPs) have been extensively studied for nanomedicine applications (Fenske and Cullis, 2008; Fenske et al., 2008). The mechanism transport across the BBB is still unclear (Agrawal et al., 2017). Initially, it was thought that the liposomes spontaneously crossed several biological membranes including BBB by electrostatic interactions. However, this theory was replaced by the possible mechanism through active transport carriers by transcytosis or a specific receptor (Spuch and Navarro, 2011). The latter hypothesis is more consolidated due to the binding of specific molecules, such as glucose and glutathione, on liposome surfaces that are able to overcome the BBB promoting the liposome translocation (Noble et al., 2014).

**Figure 2:**
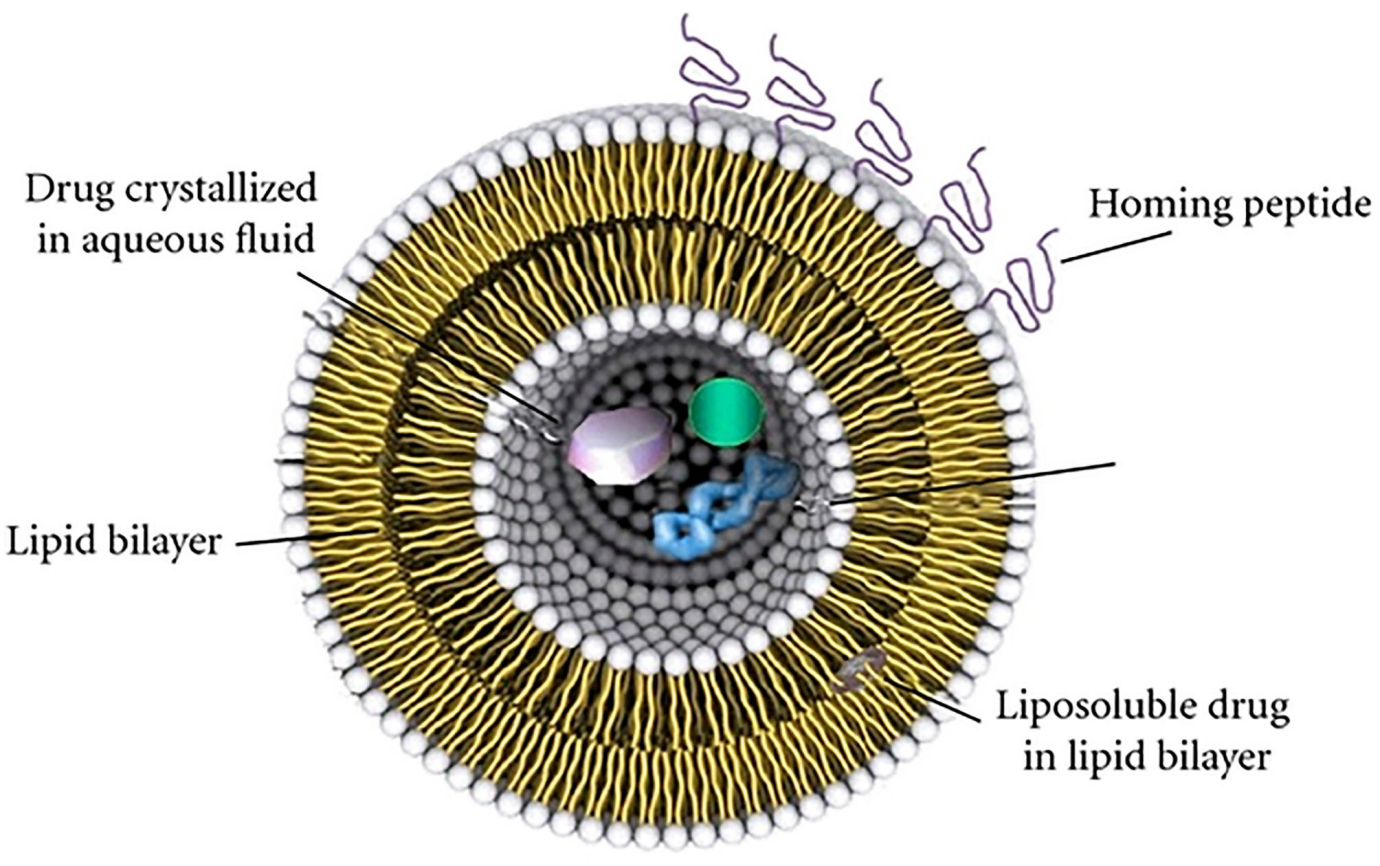
Representation of fundamental architecture of LPs. Reproduced with permission from Bitounis et al. (2012).

Their biochemical architecture (Figure 2) is similar to the human cellular membrane; in fact, it is formed by phospholipid double layers that enclose an aqueous core. In these artificial vesicles, with spherical shape, lipophilic and hydrophilic drugs can be encapsulated: instead of lipophilic molecules only confined into phospholipidic bilayers, hydrophilic molecules can be loaded both inside the aqueous cores of LPs and in the external water phase. The encapsulation efficiency is almost equal to the loading rate in the case of lipophilic molecules, whereas it depends on composition and the LP synthetic approach method in the case of hydrophilic drug (Johnsson and Edwards, 2003). However, in any case it was found that drug toxicity is strongly reduced following internalization in the LP.

Conventionally, a first classification of LPs is based on the number of bilayers (lamellarity) and on the size of the structure (Woodle and Papahadjopoulos, 1989) because these two parameters deeply influence both the circulation half-life and the drug encapsulation efficiency. Small unilamellar vesicles (SUVs) and large unilamellar vesicles (LUVs) indicate LPs formed from a single layer with a dimension of (25 ÷ 50) nm or (200 ÷ 800) nm, respectively, while multilamellar vesicles (MLV) are referred to LPs formed by concentric multiple lipid layers, separated from each other by an aqueous solution. Most specific classifications regard the chemical composition, lipid organization, surface charge, or preparation methods (Euliss et al., 2006; Bozzuto and Molinari, 2015).

For the drug delivery aim, the LPs appear a promising system, not only for their ability to protect their cargo to enzymes degradation phenomenon, but also for their unique properties, including low toxicity, flexibility, biocompatibility, biodegradability, and non-immunogenicity. Unfortunately, LPs suffer of some limitations for specific therapeutic applications, namely shelf life and the drug-loading efficiency. In the last decades, several efforts have been made toward the ingegnerization of optimal LPs, and new categories of liposomal carriers were obtained.

Solid lipid nanoparticles (SLN) have established themselves as an alternative system to conventional LPs, due to significant advantages: controlled and targeted drug release, high stability, and effective encapsulation efficiency of drugs. The SLNs structures consist of a solid lipid matrix (tri-, di-, and mono-glycerides, fatty acids, steroids, or waxes) that outlines an aqueous volume in which lipophilic and hydrophilic molecules can be internalized (Uner and Yener, 2007). However, some of the drugs could flow out during the transport following the natural recrystallization process of the SLN shell, formed by a single lipid. For that reason, the delivery efficacy results are jeopardized by the SLN architecture (Blasi et al., 2007).

With the aim to overcome these drawbacks, the nanostructured lipid carriers (NLC) were conceived. The matrix of the NLC structure is composed by a combination of solid and liquid lipids. This latter induces the nanostructuring process into the inner lipid shell. These results allow the increase of the volume available for drug loading and the boost of a more stable lipid configuration (Iqbal et al., 2012; Beloqui et al., 2016).

Several synthetic routes have been proposed to obtain SLN and NLCs; among these, high-pressure homogenization (HPH) and microemulsion are the most efficient. The microemulsion technique requires the use of hazardous toxic organic solvents; for this reason, the lipidic systems obtained through this procedure are unsuitable for medical purposes. In this perspective, the use of HPH procedure is preferred; however, this technique permits the internalization only of thermostable drugs (Das and Chaudhury, 2011).

The SLN/NLCs systems have been largely employed *in vivo* for the delivery of several kinds of drugs, by intranasal, intragastric, oral, and intravenous administrations. In order to optimize the carriers for dermal delivery, a new class of LPs has been proposed by Touitou and co-workers (Touitou et al., 2000). They modified LPs, adding ethanol, in order to obtain high efficiency in the delivery of different drugs through the skin.

**Figure 3:**
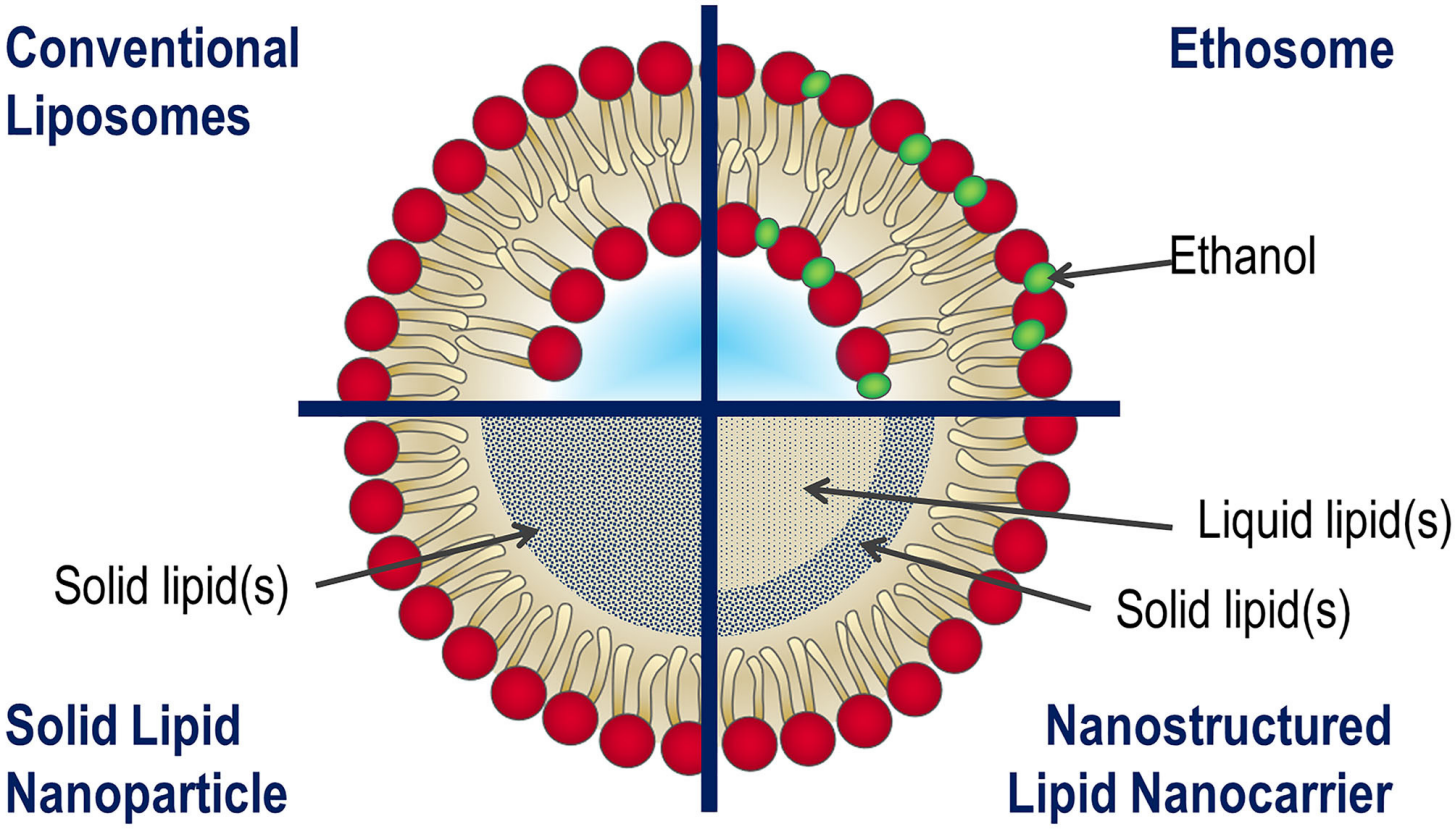
Schematic representation of main structure of LPs, ethosome, solid lipid nanoparticles (SLNs), and nanostructured lipid carriers (NLCs).

These modified LPs (Figure 3), called ethosomes, are lipid vesicular systems composed primarily of phospholipids, water, and ethanol. This latter, added in high concentration (20 ÷ 45 %), interacts with the polar heads of the phospholipids; finally, it is inserted in the lipid stratum corneum, lowering the melting-point of lipidic bilayer. This last phenomenon makes them to be more fluid and permeable for the cell membrane. In addition, in comparison with LPs, ethosomes have small dimensions due to the negative superficial charge induced by ethanol inclusion.

The improved transport mechanism of bioactive molecules through the skin can be applied to the drug delivery to the CNS; in fact, the characteristics of the ethosomes could be useful to promote the BBB penetration.

Therefore, the improvement of the liposomal delivery systems has required the optimization of the physiochemical properties of the liposomal surface in relation to the chemical nature of entrapped drug molecules.

## Conventional Therapeutic Approach in Liposomal Formulation Against ND Progression

Currently, the treatment of ND pathologies is still not available; however, many molecules and compounds can mitigate the symptoms and slow down the ND progression (Duraes et al., 2018). The first therapeutic approaches were based on the administration of small hydrophilic molecules (levodopa, rivastigmine, tacrine, donepezil), which can penetrate the BBB through the lipid-mediated diffusion process, exerting neuroprotective effects in several ND models. In recent years, the attention of medical and scientific communities has been focused on natural compounds, to overcome the side effects induced by chemical drug treatments against ND disorders. Due to anticholinesterase and the antioxidative and anti-inflammatory outcomes of different phytochemicals, these could be promising therapeutic agents against ND progression. In detail, the neuroprotective and anti-protein aggregation effects induced by curcumin, quercetin, and resveratrol were extensively investigated.

Despite numerous therapeutic agents showing promising results for ND treatments, the experimental evidences obtained *in vivo* fell short of expectations; in fact, the efficacy of all these molecules is strongly limited due to the low bioavailability and low penetration capability in BBB. In addition, the phytochemicals compounds are poorly water and blood soluble and highlighted a short biological half-life as well as low uptake in the brain (Darvesh et al., 2012; Yang et al., 2014; Kulkarni and Canto, 2015; Tellone et al., 2015; Ay et al., 2017).

The advances in the nanotechnological field have enabled the development of engineered nanocarriers to deliver drugs and to release them in a controlled manner in specific body compartments. In particular, the LPs have become the best theranostic system for anti-ND molecules, since they allow them to overcome their inherent limitations (Table 1).

**Table 1:** The table summarizes the main molecules currently used against neurodegenerative diseases.

**Drug**	**Bioactivities**	**Limits**	**Liposomal-based carriers**	***In vivo* model**	**Administration route**	**Improvements**	**References**
Rivastigmine	Acetylcholinesterase inhibitor	- Low penetration capability across BBB - Low bioavailability	Conventional multilamellar LPs	Wistar rats	Intranasal	LPs increase drug accumulation rate in brain	(Arumugam et al., 2008)
			Electrosteric stealth (EES) LPs	Rabbits	Intranasal	Bioavailability of drugs increases in plasma and brain compartments	(Nageeb El-Helaly et al., 2017)
			LPs and Cell penetrating peptides (CPP) LPs	Murine	Intranasal	LPs and CPP-LPs enhance the permeability across BBB	(Yang et al., 2013)
Donepezil	Acetylcholinesterase inhibitor	- Low bioavailability - Low BBB permeation	LPs	Wistar rats	Intranasal	LP formulation increases the brain bioavailability	(Al Asmari et al., 2016)
Curcumin	Inhibitor of amyloid protein aggregation	- Low water solubility - Low bioavailability	LPs	Wistar-Bratislava albino rats	Intravenous	Increased bioavailability and efficacy of this compound	(Bulboaca et al., 2018)
						Augmented shelf life	
			LPs	Rats	Intranasal	Considerable suppression of cytokine levels	(Sokolik et al., 2017)
						Improved efficacy in terms of cognitive responses	
			Nanosized LPs and nanosized LPs functionalized with anti-Tf antibody	*Ex vivo* human brain tissues	—	Highest therapeutic efficacy	(Mourtas et al., 2014)
						Increased permeation capability, particularly in the case of Anti-Trf LPs	
			SLN and NLC	Rats	Intravenous	Increased drug accumulation in brain tissue, especially in the NLC case	(Sadegh Malvajerd et al., 2019)
						Reduced inflammatory state respect free drug administration	
			Liposomes with WGA (curcumin combined with NGF and/or CL)	Wistar rat	Intraperitoneal	Improved permeation rate across BBB	(Kuo et al., 2017)
						Inhibition of amyloid plaques formation	
						Cholinergic activity stimulation in hippocampus area	
Quercetin	Antioxidative ability Ameliorate cognitive and memory dysfunction	Low solubility in blood poor absorption and difficulty to pass BBB	Mannosylated LPs	Sprague Dawley rat	Carotid injection	Promoted antioxidant enzyme activities	(Sarkar and Das, 2006)
			LPs	Swiss albino rat	oral	Promoted antioxidant enzyme activities	(Ghosh et al., 2009)
			LPs	Wistar rat	intranasal	Neuroprotective action in hippocampus area	(Phachonpai et al., 2010)
						Amelioration of cognitive performances	(Terdthai et al., 2010)
			ApoE-PA-LPs	Sprague Dawley	Intravenous	Increased permeation capacity	(Kuo et al., 2018)
Resveratrol	promote the non-amyloidogenic cleavage of the amyloid precursor proteins, improves β amyloid-peptides clearance and to limit neuronal injury	Low aqueous solubility and low bioavailability	SLNs and poloxamer 188 coated SLNs	Wistar rats	oral	SLN formulations augment 8-fold the bioavailability of drug suspension	(Pandita et al., 2014)
			SLNs and NLCs	Wistar rats	intraperitoneal	Increased concentration in the brain	(Neves et al., 2013; Jose et al., 2014)
			LPs	Sprague-Dawley rats	Intravenous	Greater reduction of epileptic seizure respect to free resveratrol administration	(Wang et al., 2011)
			LPs	Sprague–Dawley rats	Intravenous	Higher reduction of ROS level and epileptic events in liposomal formulation case	(Ethemoglu et al., 2017)
Levodopa (SHM)	precursor of dopamine	- Cytotoxicity (ROS productions) - Dyskinesia - Low penetration capability across BBB	Chitosan coated LPs	Sprague-Dawley rats	oral	Increased drug accumulation rate in brain	(Cao et al., 2016)
						Reduced dyskinesia outcome	
			Superficial charged LPs	Sprague-Dawley rats	Carotid arteries injection	charged LPs more actively interact with BBB	(Joshi et al., 2014b)
						charged LPs better penetrated BBB respect neutral liposomes	
						cationic LPs accumulation rate in brain is highest respect to anionic LPs	

*The limits of the treatments are reported as well as the advantages of their administration in liposomal formulation*.

In this section, we describe how LP-based formulations improve the delivery to the brain of conventional therapeutic drugs.

## Solubility, Bioavailability, and Stability

The bioavailability of given administered drug depends by several factors, such as the aqueous or blood solubility, BBB permeability and metabolic stability (Helen Chan and Stewart, 1996). Rivastigmine and Donepezil are small hydrophobic molecules largely used in Alzheimer's treatment, because they act as acetylcholinesterase inhibitor, preventing the acetylcholine degradation and increasing its availability at the synapses (Di Stefano et al., 2011).

In order to improve the bioavailability of these drugs: Nageeb El-Helaly et al. (2017) fabricated electrosteric stealth (ESS) LPs in which rivastigmine was encapsulated; meanwhile, Al Asmari et al. (2016) prepared a new liposomal formulation using 1,2-distearyl-sn-glycero-3-phosphocholine, cholesterol, and polyethylene glycol to entrap Donepezil. In details, Nageeb El-Helaly and co-workers (Nageeb El-Helaly et al., 2017) obtained an augmented stability by steric hindrance due to addition of didecyldimethyl ammonium bromide and 1,2-distearoyl-sn-glycero-3-phosphoethanolamine-N-(methoxy (polyethyleneglycol)-2000) to l-α-lecithin from soybean; consequently, the *in vivo* studies performed on 36 rabbits showed that the bioavailability of rivastigmine strongly increased in plasma and brain compartments after intranasal injection. Analogously, the Donepezil bioavailability resulted two times higher in the brain, after intranasal administration, compared to free drug in plasma (Al Asmari et al., 2016).

**Figure 4:**
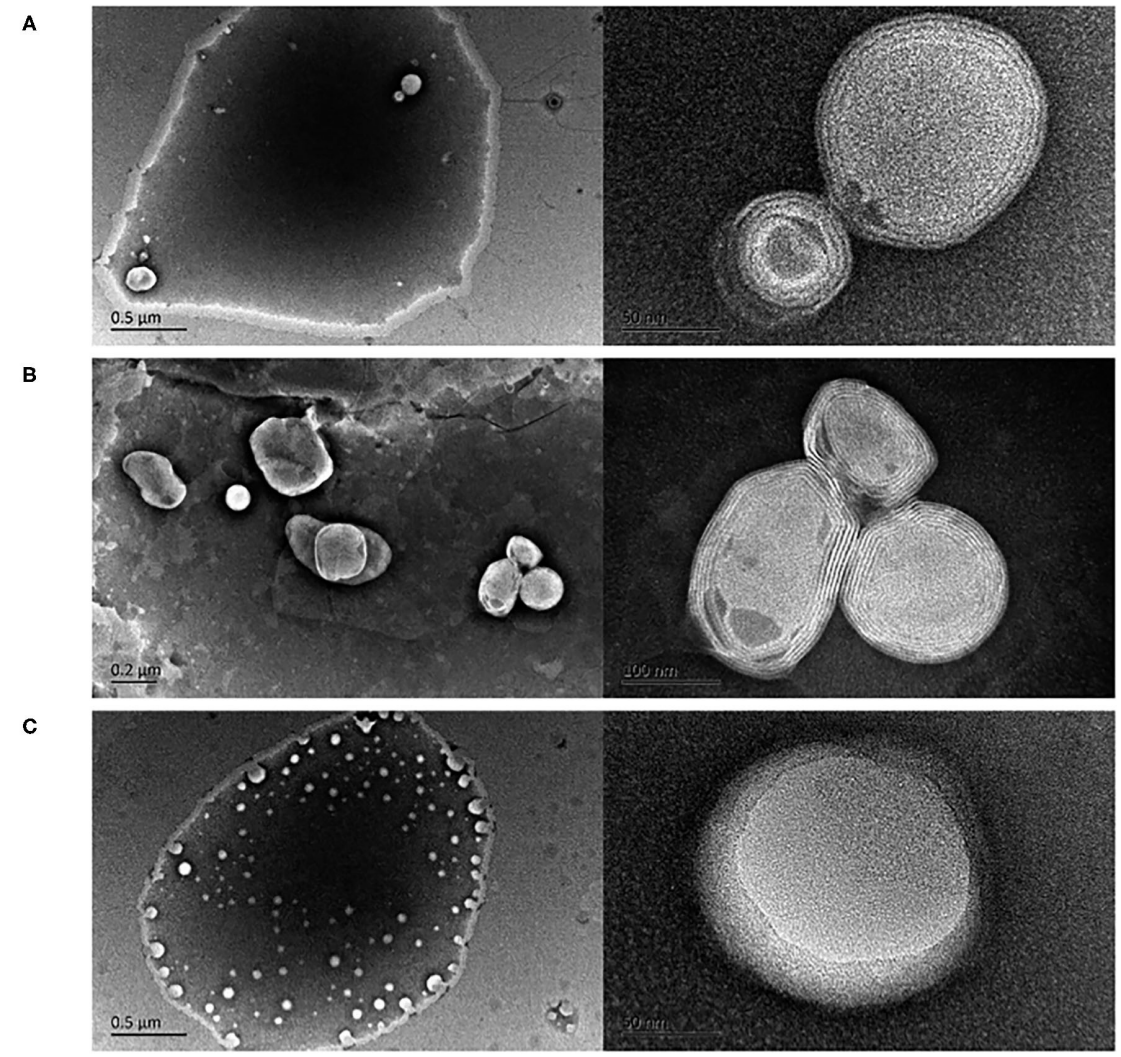
Transmission electron microscopy images of curcumin LPs produced by the **(A)** pH-driven method, **(B)** thin film method, and **(C)** ethanol injection method. Reproduced from Cheng et al. (2017) with permission of The Royal Society of Chemistry.

Among phytochemical compounds, curcumin induces neuroprotective and anti-protein aggregation effects. The low water solubility and bioavailability can be enhanced by internalization into liposomal carriers: Cheng et al. (2017) optimized a pH-driven method to prepare curcumin-loaded LPs (Figure 4), testing their bioavailability by the reproduction of gastrointestinal tissue. They found an increased bioavailability (more than twofold) with respect to curcumin solution.

Bulboaca et al. (2018) investigated the therapeutic potential of curcumin in addition to the traditional sumatriptan treatment for migraine disease using an *in vivo* rat model, demonstrating that, after intravenous administration, the shelf life of curcumin largely increased when it was encapsulated in liposomal vectors.

The polyphenolic compound resveratrol (3,4,5-trihydroxystilbene) gained increased attention as a therapeutic agent against ND diseases, due to their ability to promote the non-amyloidogenic cleavage of the amyloid precursor proteins, to improve β amyloid-peptide clearance and to limit neuronal injury (Li et al., 2012). As well as other natural compounds, the encapsulation in liposomal systems augments the drug effectiveness.

The enhancement in resveratrol bioavailability was demonstrated by Neves and collaborators by *in vitro* (Neves et al., 2013) and *in vivo* experiments (Jose et al., 2014), loading it in SLNs and NLCs. The *in vivo* biodistribution study using Wistar rats demonstrated that SLN could significantly increase the brain concentration of resveratrol (17.28 ± 0.6344 μg/g) as compared to non-liposomal formulation (3.45 ± 0.3961 μg/g). In pharmacokinetic studies conducted in male Wistar rats, Pandita et al. (2014) showed that the oral bioavailability of resveratrol was increased 8.035-fold when loaded in stearic acid-based SLNs stabilized by mixture of surfactants PHOSPHOLIPON® 90G/poloxamer 188.

In light of these experimental evidences, it is undeniable that encapsulation in liposomal carriers may offer interesting opportunities to enhance the delivery of anti-ND drugs.

## Therapeutic Efficacy and Drug Toxicity Reduction

The encapsulation of anti-NDs drugs in liposomal-based carriers permits to minimize their adverse effects and simultaneously to enhance their therapeutic action.

Among small hydrophobic molecules, Levodopa (L-dopa, L-3, 4-dihydroxyphenylalanine) is the most used molecules in ND treatment; in particular, in the last 4 decades Levodopa (L-DOPA)-based therapy become the golden standard therapy against Parkinson disease (Poewe et al., 2010). After oral administration, <10% of L-DOPA dose reaches the CNS, while the remaining 90% tends to accumulate into the liver and muscles, inducing dyskinesia (Dodel et al., 2001). These adverse effects can be reduced loading L-DOPA in chitosan-coated nano-LPs. Chitosan is a natural hydrogel cationic polysaccharide of co-polymers glucosamine and N-acetyl glucosamine obtained by alkaline deacetylation of chitin that is constitutive of crustacean shells. In addition, it shows a positive charge that can be exploited to form polyelectrolyte vesicular complexes able to act as delivery systems (Elieh-Ali-Komi and Hamblin, 2016). Cao and colleagues performed *in vivo* studies on rats, demonstrating that the abnormal involuntary movement rate significantly decreased in rats treated with chitosan-coated L-DOPA NLs than those who received free L-DOPA (Cao et al., 2016).

In the case of phytochemical compounds, the ND synthoms became more attenuated after the administration of curcumin and quercetin encapsulated in liposomal carriers with respect to free formulations. In details, *in vivo* experiments conducted on a rat model by Sadegh Malvajerd et al. (2019) showed a reduction in inflammatory state due to increased anti-oxidant activity after intravenous administrations of encapsulated curcumin into SLN and NLC carriers with respect to free drug; in addition, the pharmacokinetic studies demonstrated that the NLC option ensures higher accumulation into rat brain. Another interesting approach was suggested by Kuo et al. (2017) proposing the combined use of curcumin (CRM) and nerve growth factor (NGF) conjugated with cardiolipin (CL) to treat Alzheimer disorders. In details, the human neuroblastoma (SK-N-MC) cell line insulted with β-amyloid peptide (Aβ) shows how treatment with curcumin CRM-CL LPs prevented the neurodegeneration process through the inhibition of the p38, JNK, and tau protein phosphorylation. Meanwhile, the exposure to NGF-CL/LIP up-regulated the expression of the p-TrkA and p-ERK5, responsible to ensure neuronal viability. The effects of the combined use of these LPs were assessed in Wistar rat models, after intraperitoneal injections. To improve the permeation rate across the BBB, CRM-CL and CRM-CL LPs were implemented with germ agglutinin (WGA). The combined action of WGA-grafted CRM-CL and CRM-CL strongly affected the Aβ plaque formation, increasing the percentage of healthy neurons and promoting the cholinergic activity in the rat hippocampus.

Sokolik et al. compared the efficacy of intranasal administrated curcumin against Alzheimer's disorder in soluble form with liposomal formulations. After neuroinflammatory quantification analysis, they performed cognitive tests on rats to evaluate the impact of curcumin exposure on mnestic functions. They evaluated the impact on the angiotensin converting enzyme activity and cytokine productions in the damaged rat brain. Treatment with CLs reduced cytokines (interleukin-1β, interleukin-6, interleukin-10, tumor necrosis factor α) and angiotensin expression in cerebral cortex and hippocampus area. Finally, the cognitive tests show a reduction in the latent period of conditioned reflex reactions (Sokolik and Shulga, 2016; Sokolik et al., 2016, 2017).

Sarkar and Das have reported that, conversely to free formulation, quercetin loaded in mannosylated LPs and administrated via carotid injection in male Sprague-Dawley rats, in which the ischemia event was induced, meaningfully sustained antioxidant enzyme activity, preventing edema formation (Sarkar and Das, 2006). In addition, Das et al. demonstrated the oral administration of LP-encapsulated quercetin enhanced the antioxidative enzyme activity in adult female Swiss albino rat brains neurodamaged after arsenic exposure (Ghosh et al., 2009). Phachonpai and colleagues (Phachonpai et al., 2010) treated male Wistar rats, used as Alzheimer's disease model, with quercetin in LPs via intranasal administration. After treatment (0.5 mg/daily for 2 weeks), the neuroprotective quercetin action was recorded: the activities of superoxide dismutase, catalase, and glutathione peroxidase increased while the malondialdehyde levels were decreased. The reduction of oxidative stress confirmed the effectiveness of quercetin against Alzheimer's disease. The extension of the time treatment until 4 weeks increased the cholinergic neuron viability in hippocampus and ameliorated cognitive performances (Terdthai et al., 2010).

In the last years, the combination of resveratrol in LPs was explored as potential strategy for ND treatment. Ethemoglu et al. (2017) demonstrated how the resveratrol administration influenced the incidence of epileptic events in rats, in which brain ND disorder was induced by penicillin administration. The treatment with resveratrol reduced the epileptic activity, probably due to the reduction of oxidative stress at the cellular level. This rate further decreased when resveratrol was encapsulated in liposomal carriers.

Wang et al. (2013) assessed the impact of resveratrol derived from *Polygonum cuspidatum* on rats in which Parkinson's outcome was induced. After 14 days of oral treatment, the apoptosis rate of nigral tissue cells and ROS level was decreased while the antioxidant capability increased. These effects were strongly augmented when resveratrol was administered in liposomal vectors, probably due to the enhanced bioavailability.

## Permeability

The main limitation of anti-ND drug efficacy is their poor BBB penetration capability, in fact <98% of the small molecules and nearly 100% of large molecules are able to reach the brain. The encapsulation in liposomal-based carriers can enhance the bioavailability in brain of therapeutic agents; for example Aramugam et al. (2008) treated Wistar rat with rivastigmine encapsulated in conventional multilamellar LPs: after intranasal injection, the rivastigmine level reveled in brain tissue resulted in higher respect to free drug. However, in order to improve the uptake of anti-ND drugs, active targeting strategies have been developed. The chemical modification of LP surfaces essentially consists in attaching ligands which interact with proteins expressed by BBB.

Yang et al. (2013) produced CCP LPs using cell-penetrating peptide (CPP), with the aim to enhance the brain distribution of rivastigmine. In detail, they proceeded by an intranasal administration in order to minimize the collateral effects (including hepatic first pass metabolism and gastrointestinal adverse outcomes), enhancing the permeability of the CPP-LP complex across both the nasal olfactory pathway and the murine BBB. Relative to the extended release and absorption, an intense inhibition of acetylcholinesterase and butyrylcholinesterase activities was detected.

Among receptor-mediated transcytosis, the transferrin (Tf) receptors are the one of the most explored as targets of the drug delivery system (Sharma et al., 2016). In particular, several *in vitro* and *in vivo* studies were performed in order to improve the targeting efficacy of therapeutic molecules across BBB (Pulgar, 2018).

Mourtas et al. (2014) produced a curcumin derivative in nanosized LPs functionalized with the anti-Tf antibody to direction them against Aβ protein in amyloid plaques. The potential efficacy of curcumin-derivative LPs and curcumin-derivative anti-Tf LPs was assessed in *ex vivo* experiments performed on post-mortem extracted brain samples from patients affected by Alzheimer disease. Both LP types exhibited high binding affinity with Aβ proteins; indeed, the thioflavin assay confirmed their ability to slow down the Aβ1-42 peptide aggregation. Although the efficacy of examined carriers was comparable, the anti-Tf receptor-decorated LPs strongly enhanced the brain permeation.

With the aim to improve the efficacy of the neuroprotectant ZL006 against ischemic stroke, Wang and colleagues (Wang et al., 2015) encapsulated it in modified LPs with HAIYPRH (T7) peptide, which directly interacts with TfR. The *in vivo* experiments performed on rat models (Sprague-Dawley and ICR mice) revealed that the T7 functionalization considerably improved the penetration across the BBB. This implied a reduced infarct volume and enhanced neurological deficit with respect to the untargeted LPs or SKfree ZL006.

The Tf functionalization is currently used to improve BBB targeting and transcytosis. In order to enhance the neuronal transfection in gene therapy, Dos Santos Rodrigues et al. (Dos Santos Rodrigues et al., 2018) conceived LPs with double functionalization: Tf and penetratin (Pen); the latter enabled overcoming receptor saturation and increasing the number of internalized LPs. Hydrophilic plasmid for β-galactosidase (pβgal), used as a gene model, were loaded in Pen-Tf LPs in order to explore the efficacy in gene therapy applications. After injection in C57BL/6J mice, the plasmid biodistribution was quantified in different tissues: the liposomal formulation involved a significant increase in βgal amount in brain, liver, and kidneys with respect to the endogenous levels. In addition, the Pen-Tf functionalization improved the transfection capability only in the brain.

α-Mangostin is a polyphenolic xanthone, largely used in medicine; recently, it was demonstrated its capability to slow down AD progression (Wang et al., 2012); nevertheless, its efficacy is limited by the low penetration capability across the BBB. Moving from these evidences, Chen and colleagues (Chen et al., 2016) modified LPs with Tf in order to enhance the penetration rate of α-Mangostin in brain. α-M was intravenously administrated (5 mg/kg) into the Sprague Dawley rats in different formulations: α-M solution, α-M LP, and Tf(α-M) LP. The pharmacokinetic experiments revealed that encapsulation in Tf LPs augmented the α-M bioavailability in plasma and drug accumulation rate in brain tissue. The authors proposed Tf-LPs as a promising drug delivery system, demonstrating their ability to improve the brain permeation of α-M; however, α-M distribution quantification in different body compartments showed that Tf(α-M) LPs tended to accumulate also in liver tissue; this is due to the expression of TfR on hepatocytes.

The fact that Tf receptors were expressed on several cellular types, including hepatocytes, monocytes, erythrocytes, intestinal cells, epithelial cell of choroid plexus, and neuron besides BBB, represents an important issue in targeting of delivery systems. In addition, the efficiency of the Tf targeting could be dramatically compromised by the physiological presence of high levels of endogenous Tf in the plasma, which saturated the TfR endothelial cells (Sharma et al., 2016).

Like TfR, low-density lipoprotein (LDL) receptors, involved in the specific BBB transport mechanisms, could be exploited in the increase of the drug rate permeation in brain. In particular, human apolipoprotein E (apoE) plays a key role in lipid transport in the CNS (Fan et al., 2001; Hatters et al., 2006). In addition, biological membranes contain a great variety of phospholipids; one among them is phosphatidic acid (PA), which has a special attraction for Aβ and may help rescue neurons from Aβ-induced toxicity. Drug carriers associated with PA and ApoE may improve the affinity to binding to Aβ *in vitro* (Re et al., 2011; Salvati et al., 2013).

Starting from these evidences, Bana et al. (2014) used a Balb/c mice model to test the uptake in brain of modified LPs mApoE-PA-LPs, obtained by functionalizing PA and a synthetic peptide (mApoE; CWGLRKLRKRLLR) that includes the apolipoprotein-E receptor-binding domain. The biodistribution experiments, performed after intravenous administration, demonstrated that mApoE-PA-LPs were more capable of penetrating the BBB respect to PA-LPs.

The apolipoprotein E (ApoE) conjugated with PA was also used by Kuo and co-worker (Kuo et al., 2018) to functionalize LPs (ApoE-PA-LP) able to deliver quercetin and rosmarinic acid to brain, in order to inhibit β-amyloid (1–42) (Aβ1-42) aggregation. The permeation capacity strongly increased in functionalization case respect to free drugs and drugs encapsulated in undecorated LPs. The administration of quercetin and rosmarinic acid in ApoE-PA-LP formulation induced in Aβ-insulted SD rats the diminishment of the acetylcholinesterase activity and the reduction in Aβ plaque formation.

Recently, other potential targeting molecules have been explored to improve the drug internalization exploiting the receptor-mediated transcytosis process. In detail, the nutrient transporters can be used for brain delivery. Among these, glutathione (GSH) is an endogenous tripeptide able to penetrate the BBB as sodium-dependent transporter; this latter is preferentially expressed in CNS and BBB of all mammalian species (Smeyne and Smeyne, 2013; Kuo et al., 2018).

Rip and co-workers fabricated PEGylated LPs with GSH, and they investigated the potential improvement in brain uptake of drug encapsulated in this carrier; for this purpose the authors loaded a fluorescent tracer (carboxifluorescein) in the PEG-GSH modified LPs (Rip et al., 2014). Quantification of carboxifluorescein in brain was obtained by microdialysis experiment on Wistar rats, in which this fluorescent molecule was intravenously administrated in three different forms: free, non-targeted PEG LPs, and GSH-PEG LPs. The results showed that the carboxifluorescein level in brain increased in liposomal formulation; in particular, the GSH-PEG LP option made this level fourfold higher with respect to undecorated PEG LPs. The efficiency of GSH-PEG LP delivery in the brain was also tested for ribavirin drug (Rip et al., 2010; Maussang et al., 2016). The concentration of drug in brain tissue increased in modified LP formulation in Wistar rats after administration by intravenous route; the permeation rate beyond the BBB became proportional to the amount of GSH coating (ranging from 0 to 2%).

In addition, in literature several works reported how the GSH-PEG LP formulation improves in brain delivery of the opioid peptide DAMGO (H-Tyr-d-Ala-Gly-MePhe-Gly-ol) (Lindqvist et al., 2013), the anti-amyloid single-domain antibody fragment (Rotman et al., 2015), flucytosine (Salem et al., 2015), methylprednisolone hemisuccinate (Kanhai et al., 2018), etc.

Another promising strategy to improve penetration capability of anti-ND drug is based on the use of cationic polyelectrolyte in LP preparation. Cationic LPs (CLs) can penetrate the BBB through adsorptive-mediated endocytosis process (Herve et al., 2008). This occurs because the surface of CLs exhibits a positive charge at physiological pH; thus, it electrostatically interacts with the polyanions present in the BBB (Miller et al., 1998).

This event was examined *in vivo* on Sprague–Dawley rats, in which cationic, anionic, and charge-neutral LPs were directly injected into carotid arteries. Joshi et al. reported that superficial charged LPs more actively interact with BBB endothelial cell respect to neutral LPs; in particular, the authors recorded the highest cationic concentration with respect to the anionic LPs into the brain tissue (Joshi et al., 2014a,b; Joshi et al., 2015).

Migliore et al. (2010) stated that the use of CLs as a delivery system enhanced the brain uptake of drug administered by intranasal route in Sprague–Dawley rats.

Recently, another work (Dhaliwal et al., 2020) reported how it is possible to take advantage of cationic LPs to improve the mRNA delivery. After optimization of the liposomal formulation, mRNA was administered by intranasal route to CD-1 mice. Through the GFP and luciferase reporter systems, the authors have quantified the mRNA biodistribution *in vivo*. The findings demonstrated the cationic LPs improved the penetration rate of mRNA and its accumulation into specific cerebral areas (cortex, striatum, and midbrain).

Although it was demonstrated that the CL formulation enhanced the anti-ND drug penetration rate in the brain, it must be admitted that this rate is limited by non-specific uptake phenomena in peripheral tissues. In addition, the superficial charge of LPs could be reduced by interaction with serum protein before reaching the BBB (Tagalakis et al., 2014).

These issues could be overcome by increasing the amounts of use of CLs, but keeping in consideration their cytotoxic potential.

## Administration Routes

The main challenge in the treatment of CNS diseases is represented by the presence of the BBB, which makes complicated the delivery of therapeutic agents into the brain. At first, invasive approaches have performed in order to overcome the BBB, including the neurosurgery-based cerebral infusions or implants and the physical or chemical disruption of the BBB to permit the penetration of drug via osmotic shift (Chen et al., 2004). However, these techniques are highly dangerous carry risks of infection and brain tissue damage, and are able to induce chronic neuropathological effects in treated patients (Chen et al., 2004). In recent decades, several non-invasive strategies, based on physiological brain transport mechanisms, have been developed.

As already described in this review, the drug loading in LPs increases its own uptake in the brain; later on, once the importance of LP usage was proven, various studies have tried several routes of administration for finding out if they are more or less adequate.

The intravenous routes are certainly the most common, preferred to the oral route, because in this latter case there is a loss in terms of drug percentage in the gastrointestinal ambient: drug is degraded by the stomach fluids and poorly absorbed by the intestine, while increasing the hepatic and renal load; although in different reported works, it has been proven that the drug loading in LPs prevent the drug degradation. Although the intravenous injection is the most applied, the interest in alternative parenteral routes increased, for finding new direct administrations to the BBB. Among these, the intranasal way gained a noticeable increase by the scientific community, due to its ability to let the drug reach the brain via different pathways: direct ones, through olfactive epithelium, thanks to the presence of olfactory nerves; or indirect ones, through the respiratory epithelium first and the circulatory system after (Hong et al., 2019).

In the last decade, there is a growing interest of administering them via intradermal routes. In this perspective, ethosomes represent the better choice among liposomal systems, due to their ability to increase the drug permeation across the derma. For instance, Ropinirole hydrochloride is commonly used in Parkinson disease. With the aim to evaluate the possibility to administrate Ropinirole hydrochloride via transdermal route, Mishra et al. (2013) synthetized different ethosomal carriers, varying ethanol and lecithin concentrations. The experimental *in vivo* study showed that RHC encapsulated in ethosomes (30 and 4% w/v of ethanol and lecithin, respectively) increased their blood circulation time, becoming comparable to the oral administration case.

Flurbiprofen is largely used in ND treatments for its anti-inflammatory effect. The permeation of ethosomal–flurbiprofen (EF) formulations was assessed in the albino rat model (Paliwal et al., 2019). The reported results showed a high encapsulation drug efficiency (95 %); in addition, the permeation efficacy was equal to (82.56 ± 2.11) g/cm^2^ in 24 h, and transdermal flux was found as 226.1 μg/cm^2^/h.

Ligustrazine phosphate (LP) is an antioxidant used for the Alzheimer disorder. Shi and co-workers (Shi et al., 2012) optimized the ethosome-based carrier for LP transdermal delivery; in addition, they evaluated the therapeutic impact on rats, in which amnesia disease was induced by scopolamine. Experimental evidence indicated that the penetration ability of the LP ethosomal system was strongly higher than the LP in aqueous solution. Moreover, the LP ethosomal treatment normalized the levels both of antioxidant enzymes and the lipid peroxidation in a healthy rat model.

These *in vivo* evidences suggested the goodness of ethosome-based strategy; nevertheless, for ND disease, only a few studies explored the potential of dermal administration ethosome-mediated.

## Conclusions and Perspective

In the last decades, many efforts have been made to develop an optimal therapeutic strategy against neurodegenerative disorders due to their dramatic incidence worldwide. In this perspective, several new molecules including phytochemical extracts were investigated along with the conventional drugs. Unfortunately, their therapeutic potential was strongly compromised owing to the presence of high selective barrier (BBB) that protects the brain from treatment (CNS).

The effectiveness of drugs against ND diseases increases when they are encapsulated into liposomal-based vectors.

Generally, LPs represent a promising platform for theranostic medicine purposes, thanks to their size, biocompatibility, high biological affinity, immunogenicity, biodegradability, and low toxicity. In addition, LPs have a good ability to deliver both hydrophobic and hydrophilic molecules through BBB.

In this review article, the recent state of the art about the use of liposomal-based carriers for anti-neurodegenerative drug delivery and the *in vivo* applications are extensively examined. In the reported researches, the enhanced efficacy of drug following liposomal encapsulation has been demonstrated, in terms of improved bioavailability, stability, and permeation capability across BBB.

Albeit a great deal of progress has been made in liposomal formulations and their efficiency was demonstrated in several *in vivo* studies, some issues are still present.

The scientific community is called to perform further clinical trials in order to evaluate the LP accumulation effects induced by long-term exposure and, simultaneously, to optimize the ingegnerization of these carriers in the human body.

## Author Contributions

MC and VDM: conceptualization and writing (original draft preparation). MC, VDM, SL, and RR: review and editing manuscript. SL and RR: funding acquisition.

## Conflict of Interest

The authors declare that the research was conducted in the absence of any commercial or financial relationships that could be construed as a potential conflict of interest.

## References

[B1] Abbott N. J. Patabendige A. A. Dolman D. E. Yusof S. R. Begley D. J. (2010). Structure and function of the blood-brain barrier. Neurobiol. Dis. 37, 13–25. 10.1016/j.nbd.2009.07.030 19664713

[B2] Abbott N. J. Romero I. A. (1996). Transporting therapeutics across the blood-brain barrier. Mol. Med. Today 2, 106–113. 10.1016/1357-4310(96)88720-X 8796867

[B3] Abeliovich A. Gitler A. D. (2016). Defects in trafficking bridge parkinson's disease pathology and genetics. Nature 539, 207–216. 10.1038/nature20414 27830778

[B4] Agrawal M. Ajazuddin T. D. K Saraf S. Saraf S. Antimisiaris S. G. Mourtas S.. (2017). Recent advancements in liposomes targeting strategies to cross blood-brain barrier (bbb) for the treatment of alzheimer's disease. J. Control. Release 260, 61–77. 10.1016/j.jconrel.2017.05.019 28549949

[B5] Al Asmari A. K. Ullah Z. Tariq M. Fatani A. (2016). Preparation, characterization, and *in vivo* evaluation of intranasally administered liposomal formulation of donepezil. Drug Des. Devel. Ther. 10, 205–215. 10.2147/DDDT.S93937 26834457 PMC4716722

[B6] Alexander G. E. (2004). Biology of parkinson's disease: Pathogenesis and pathophysiology of a multisystem neurodegenerative disorder. Dialogues Clin. Neurosci. 6, 259–280. 22033559 10.31887/DCNS.2004.6.3/galexander PMC3181806

[B7] Angeles D. C. Ho P. Chua L. L. Wang C. Yap Y. W. Ng C.. (2014). Thiol peroxidases ameliorate lrrk2 mutant-induced mitochondrial and dopaminergic neuronal degeneration in drosophila. Hum. Mol. Genet. 23, 3157–3165. 10.1093/hmg/ddu026 24459295 PMC4030771

[B8] Arumugam K. Subramanian G. S. Mallayasamy S. R. Averineni R. K. Reddy M. S. Udupa N. A. (2008). Study of rivastigmine liposomes for delivery into the brain through intranasal route. Acta Pharm. 58, 287–297. 10.2478/v10007-008-0014-3 19103565

[B9] Ay M. Luo J. Langley M. Jin H. Anantharam V. Kanthasamy A.. (2017). Molecular mechanisms underlying protective effects of quercetin against mitochondrial dysfunction and progressive dopaminergic neurodegeneration in cell culture and mitopark transgenic mouse models of parkinson's disease. J. Neurochem. 141, 766–782. 10.1111/jnc.14033 28376279 PMC5643047

[B10] Bana L. Minniti S. Salvati E. Sesana S. Zambelli V. Cagnotto A.. (2014). Liposomes bi-functionalized with phosphatidic acid and an apoe-derived peptide affect abeta aggregation features and cross the blood-brain-barrier: implications for therapy of alzheimer disease. Nanomedicine 10, 1583–1590. 10.1016/j.nano.2013.12.001 24333591

[B11] Bartels A. L. (2011). Blood-brain barrier p-glycoprotein function in neurodegenerative disease. Curr. Pharm. Des. 17, 2771–2777. 10.2174/138161211797440122 21831040

[B12] Begley D. J. (2004). Delivery of therapeutic agents to the central nervous system: the problems and the possibilities. Pharmacol. Ther. 104, 29–45. 10.1016/j.pharmthera.2004.08.001 15500907

[B13] Begley D. J. Brightman M. W. (2003). Structural and functional aspects of the blood-brain barrier. Progress in drug research. Fortschritte der Arzneimittelforschung. Progr. Rec. Pharm. 61, 39–78. 10.1007/978-3-0348-8049-7_2 14674608

[B14] Beloqui A. Solinis M. A. Rodriguez-Gascon A. Almeida A. J. Preat V. (2016). Nanostructured lipid carriers: promising drug delivery systems for future clinics. Nanomedicine 12, 143–161. 10.1016/j.nano.2015.09.004 26410277

[B15] Bitounis D. Fanciullino R. Iliadis A. Ciccolini J. (2012). Optimizing druggability through liposomal formulations: new approaches to an old concept. ISRN Pharm. 2012:738432 10.5402/2012/738432 22474607 PMC3302123

[B16] Blasi P. Giovagnoli S. Schoubben A. Ricci M. Rossi C. (2007). Solid lipid nanoparticles for targeted brain drug delivery. Adv. Drug Deliv. Rev. 59, 454–477. 10.1016/j.addr.2007.04.011 17570559

[B17] Bozzuto G. Molinari A. (2015). Liposomes as nanomedical devices. Int. J. Nanomed. 10, 975–999. 10.2147/IJN.S68861 PMC4324542 25678787

[B18] Bulboaca A. E. Bolboaca S. D. Stanescu I. C. Sfrangeu C. A. Porfire A. Tefas L. (2018). The effect of intravenous administration of liposomal curcumin in addition to sumatriptan treatment in an experimental migraine model in rats. Int. J. Nanomed. 13, 3093–3103. 10.2147/IJN.S162087 PMC5975613 29872296

[B19] Cao X. Hou D. Wang L. Li S. Sun S. Ping Q.. (2016). Effects and molecular mechanism of chitosan-coated levodopa nanoliposomes on behavior of dyskinesia rats. Biol. Res. 49:32. 10.1186/s40659-016-0093-4 27378167 PMC4932756

[B20] Cavaco M. Gaspar D. Arb Castanho M. Neves V. (2020). Antibodies for the treatment of brain metastases, a dream or a reality? Pharmaceutics 12:62 10.3390/pharmaceutics12010062 PMC7023012 31940974

[B21] Chen C. Han D. Cai C. Tang X. (2010). An overview of liposome lyophilization and its future potential. J. Control. Release 142, 299–311. 10.1016/j.jconrel.2009.10.024 19874861

[B22] Chen Y. Dalwadi G. Benson H. A. (2004). Drug delivery across the blood-brain barrier. Curr. Drug Deliv. 1, 361–376. 10.2174/1567201043334542 16305398

[B23] Chen Y. Liu L. (2012). Modern methods for delivery of drugs across the blood-brain barrier. Adv. Drug Deliv. Rev. 64, 640–665. 10.1016/j.addr.2011.11.010 22154620

[B24] Chen Z. L. Huang M. Wang X. R. Fu J. Han M. Shen Y. Q.. (2016). Transferrin-modified liposome promotes alpha-mangostin to penetrate the blood-brain barrier. Nanomedicine 12, 421–430. 10.1016/j.nano.2015.10.021 26711963

[B25] Cheng C. Peng S. Li Z. Zou L. Liu W. Liu C. (2017). Improved bioavailability of curcumin in liposomes prepared using a ph-driven, organic solvent-free, easily scalable process. RSC Adv. 7, 25978–25986. 10.1039/C7RA02861J

[B26] Citron M. (2002). Alzheimer's disease: treatments in discovery and development. Nat. Neurosci. 5, 1055–1057. 10.1038/nn940 12403985

[B27] Combs B. Hamel C. Kanaan N. M. (2016). Pathological conformations involving the amino terminus of tau occur early in alzheimer's disease and are differentially detected by monoclonal antibodies. Neurobiol. Dis. 94, 18–31. 10.1016/j.nbd.2016.05.016 27260838 PMC4983528

[B28] Darvesh A. S. Carroll R. T. Bishayee A. Novotny N. A. Geldenhuys W. J. Van der Schyf C. J. (2012). Curcumin and neurodegenerative diseases: a perspective. Expert Opin. Investig. Drugs 21, 1123–1140. 10.1517/13543784.2012.693479 22668065

[B29] Das S. Chaudhury A. (2011). Recent advances in lipid nanoparticle formulations with solid matrix for oral drug delivery. AAPS PharmSciTech 12, 62–76. 10.1208/s12249-010-9563-0 21174180 PMC3066374

[B30] Dawson T. M. Ko H. S. Dawson V. L. (2010). Genetic animal models of parkinson's disease. Neuron 66, 646–661. 10.1016/j.neuron.2010.04.034 20547124 PMC2917798

[B31] de Boer A. G. Gaillard P. J. (2007). Drug targeting to the brain. Annu. Rev. Pharmacol. Toxicol. 47, 323–355. 10.1146/annurev.pharmtox.47.120505.105237 16961459

[B32] de Boer A. G. van der Sandt I. C. Gaillard P. J. (2003). The role of drug transporters at the blood-brain barrier. Annu. Rev. Pharmacol. Toxicol. 43, 629–656. 10.1146/annurev.pharmtox.43.100901.140204 12415123

[B33] Demeule M. Currie J. C. Bertrand Y. Che C. Nguyen T. Regina A.. (2008). Involvement of the low-density lipoprotein receptor-related protein in the transcytosis of the brain delivery vector angiopep-2. J. Neurochem. 106, 1534–1544. 10.1111/j.1471-4159.2008.05492.x 18489712

[B34] Dhaliwal H. K. Fan Y. Kim J. Amiji M. M. (2020). Intranasal delivery and transfection of mrna therapeutics in the brain using cationic liposomes. Mol. Pharm. 17, 1996–2005. 10.1021/acs.molpharmaceut.0c00170 32365295

[B35] Di Stefano A. Iannitelli A. Laserra S. Sozio P. (2011). Drug delivery strategies for alzheimer's disease treatment. Expert Opin. Drug Deliv. 8, 581–603. 10.1517/17425247.2011.561311 21391862

[B36] Dodel R. C. Berger K. Oertel W. H. (2001). Health-related quality of life and healthcare utilisation in patients with parkinson's disease: impact of motor fluctuations and dyskinesias. Pharmacoeconomics 19, 1013–1038. 10.2165/00019053-200119100-00004 11735671

[B37] Dos Santos Rodrigues B. Oue H. Banerjee A. Kanekiyo T. Singh J. (2018). Dual functionalized liposome-mediated gene delivery across triple co-culture blood brain barrier model and specific *in vivo* neuronal transfection. J. Control. Release 286, 264–278. 10.1016/j.jconrel.2018.07.043 30071253 PMC6138570

[B38] Duraes F. Pinto M. Sousa E. (2018). Old drugs as new treatments for neurodegenerative diseases. Pharmaceuticals 11:44. 10.3390/ph11020044 29751602 PMC6027455

[B39] Elieh-Ali-Komi D. Hamblin M. R. (2016). Chitin and chitosan: production and application of versatile biomedical nanomaterials. Int. J. Adv. Res. 4, 411–427. 27819009 PMC5094803

[B40] Engelhardt B. Liebner S. (2014). Novel insights into the development and maintenance of the blood-brain barrier. Cell Tissue Res, 355, 687–699. 10.1007/s00441-014-1811-2 24590145 PMC3972432

[B41] Ethemoglu M. S. Seker F. B. Akkaya H. Kilic E. Aslan I. Erdogan C. S.. (2017). Anticonvulsant activity of resveratrol-loaded liposomes *in vivo*. Neuroscience 357, 12–19. 10.1016/j.neuroscience.2017.05.026 28577913

[B42] Euliss L. E. DuPont J. A. Gratton S. DeSimone J. (2006). Imparting size, shape, and composition control of materials for nanomedicine. Chem. Soc. Rev. 35, 1095–1104. 10.1039/b600913c 17057838

[B43] Fan Q. W. Iosbe I. Asou H. Yanagisawa K. Michikawa M. (2001). Expression and regulation of apolipoprotein e receptors in the cells of the central nervous system in culture: a review. J. Am. Aging Assoc. 24, 1–10. 10.1007/s11357-001-0001-9 23604870 PMC3455648

[B44] Fenske D. B. Chonn A. Cullis P. R. (2008). Liposomal nanomedicines: an emerging field. Toxicol. Pathol. 36, 21–29. 10.1177/0192623307310960 18337218

[B45] Fenske D. B. Cullis P. R. (2008). Liposomal nanomedicines. Expert Opin. Drug Deliv. 5, 25–44. 10.1517/17425247.5.1.25 18095927

[B46] Ghosh A. Mandal A. K. Sarkar S. Panda S. Das N. (2009). Nanoencapsulation of quercetin enhances its dietary efficacy in combating arsenic-induced oxidative damage in liver and brain of rats. Life Sci. 84, 75–80. 10.1016/j.lfs.2008.11.001 19036345

[B47] Gitler A. D. Dhillon P. Shorter J. (2017). Neurodegenerative disease: models, mechanisms, and a new hope. Dis. Model. Mech. 10, 499–502. 10.1242/dmm.030205 28468935 PMC5451177

[B48] Goedert M. (2004). Tau protein and neurodegeneration. Semin. Cell Dev. Biol. 15, 45–49. 10.1016/j.semcdb.2003.12.015 15036206

[B49] Hatters D. M. Peters-Libeu C. A. Weisgraber K. H. (2006). Apolipoprotein e structure: insights into function. Trends Biochem. Sci. 31, 445–454. 10.1016/j.tibs.2006.06.008 16820298

[B50] Heemels M. T. (2016). Neurodegenerative diseases. Nature 539:179. 10.1038/539179a 27830810

[B51] Helen Chan O. Stewart B. H. (1996). Physicochemical and drug-delivery considerations for oral drug bioavailability. Drug Discov. Today 1, 461–473. 10.1016/1359-6446(96)10039-8

[B52] Herve F. Ghinea N. Scherrmann J. M. (2008). Cns delivery via adsorptive transcytosis. AAPS J. 10, 455–472. 10.1208/s12248-008-9055-2 18726697 PMC2761699

[B53] Hong S. S. Oh K. T. Choi H. G. Lim S. J. (2019). Liposomal formulations for nose-to-brain delivery: recent advances and future perspectives. Pharmaceutics 11:540. 10.3390/pharmaceutics11100540 31627301 PMC6835450

[B54] Iqbal M. A. Md S. Sahni J. K. Baboota S. Dang S. Ali J. (2012). Nanostructured lipid carriers system: recent advances in drug delivery. J. Drug Target 20, 813–830. 10.3109/1061186X.2012.716845 22931500

[B55] Johnsson M. Edwards K. (2003). Liposomes, disks, and spherical micelles: aggregate structure in mixtures of gel phase phosphatidylcholines and poly(ethylene glycol)-phospholipids. Biophys. J. 85, 3839–3847. 10.1016/S0006-3495(03)74798-5 14645073 PMC1303685

[B56] Jose S. Anju S. S. Cinu T. A. Aleykutty N. A. Thomas S. Souto E. B. (2014). *In vivo* pharmacokinetics and biodistribution of resveratrol-loaded solid lipid nanoparticles for brain delivery. Int. J. Pharm. 474, 6–13. 10.1016/j.ijpharm.2014.08.003 25102112

[B57] Joshi S. Singh-Moon R. Wang M. Chaudhuri D. B. Ellis J. A. Bruce J. N.. (2014b). Cationic surface charge enhances early regional deposition of liposomes after intracarotid injection. J. Neurooncol. 120, 489–497. 10.1007/s11060-014-1584-1 25195130 PMC4237707

[B58] Joshi S. Singh-Moon R. P. Ellis J. A. Chaudhuri D. B. Wang M. Reif R.. (2015). Cerebral hypoperfusion-assisted intra-arterial deposition of liposomes in normal and glioma-bearing rats. Neurosurgery 76, 92–100. 10.1227/NEU.0000000000000552 25525695 PMC4273869

[B59] Joshi S. Singh-Moon R. P. Wang M. Chaudhuri D. B. Holcomb M. Straubinger N. L.. (2014a). Transient cerebral hypoperfusion assisted intraarterial cationic liposome delivery to brain tissue. J. Neurooncol. 118, 73–82. 10.1007/s11060-014-1421-6 24664370 PMC4038763

[B60] Kanhai K. M. S. Zuiker R. Stavrakaki I. Gladdines W. Gaillard P. J. Klaassen E. S.. (2018). Glutathione-pegylated liposomal methylprednisolone in comparison to free methylprednisolone: slow release characteristics and prolonged lymphocyte depression in a first-in-human study. Br. J. Clin. Pharmacol 84, 1020–1028. 10.1111/bcp.13525 29385232 PMC5903232

[B61] Kisler K. Nelson A. R. Montagne A. Zlokovic B. V. (2017). Cerebral blood flow regulation and neurovascular dysfunction in alzheimer disease. Nat. Rev. Neurosci. 18, 419–434. 10.1038/nrn.2017.48 28515434 PMC5759779

[B62] Kulkarni S. S. Canto C. (2015). The molecular targets of resveratrol. Biochim. Biophys. Acta 1852, 1114–1123. 10.1016/j.bbadis.2014.10.005 25315298

[B63] Kuo Y.-C. Chen I.-Y. Rajesh R. (2018). Use of functionalized liposomes loaded with antioxidants to permeate the blood–brain barrier and inhibit β-amyloid-induced neurodegeneration in the brain. J. Taiwan Inst. Chem. Eng. 87, 1–14. 10.1016/j.jtice.2018.03.001

[B64] Kuo Y. C. Lin C. Y. Li J. S. Lou Y. I. (2017). Wheat germ agglutinin-conjugated liposomes incorporated with cardiolipin to improve neuronal survival in alzheimer's disease treatment. Int. J. Nanomed. 12, 1757–1774. 10.2147/IJN.S128396 28280340 PMC5340244

[B65] Li F. Gong Q. Dong H. Shi J. (2012). Resveratrol, a neuroprotective supplement for alzheimer's disease. Curr. Pharm. Des. 18, 27–33. 10.2174/138161212798919075 22211686

[B66] Lindqvist A. Rip J. Gaillard P. J. Bjorkman S. Hammarlund-Udenaes M. (2013). Enhanced brain delivery of the opioid peptide damgo in glutathione pegylated liposomes: a microdialysis study. Mol. Pharm. 10, 1533–1541. 10.1021/mp300272a 22934681

[B67] Lockman P. R. Mumper R. J. Khan M. A. Allen D. D. (2002). Nanoparticle technology for drug delivery across the blood-brain barrier. Drug Dev. Ind. Pharm. 28, 1–13. 10.1081/DDC-120001481 11858519

[B68] Maussang D. Rip J. van Kregten J. van den Heuvel A. van der Pol S. van der Boom B.. (2016). Glutathione conjugation dose-dependently increases brain-specific liposomal drug delivery *in vitro* and *in vivo*. Drug Discov. Today Technol. 20, 59–69. 10.1016/j.ddtec.2016.09.003 27986226

[B69] McEwen B. S. Reagan L. P. (2004). Glucose transporter expression in the central nervous system: relationship to synaptic function. Eur. J. Pharmacol. 490, 13–24. 10.1016/j.ejphar.2004.02.041 15094070

[B70] Migliore M. M. Vyas T. K. Campbell R. B. Amiji M. M. Waszczak B. L. (2010). Brain delivery of proteins by the intranasal route of administration: a comparison of cationic liposomes versus aqueous solution formulations. J. Pharm. Sci. 99, 1745–1761. 10.1002/jps.21939 19774660

[B71] Miller C. R. Bondurant B. McLean S. D. McGovern K. A. O'Brien D. F. (1998). Liposome-cell interactions *in vitro*: effect of liposome surface charge on the binding and endocytosis of conventional and sterically stabilized liposomes. Biochemistry 37, 12875–12883. 10.1021/bi980096y 9737866

[B72] Mishra A. D. Patel C. N. Shah D. R. (2013). Formulation and optimization of ethosomes for transdermal delivery of ropinirole hydrochloride. Curr. Drug Deliv. 10, 500–516. 10.2174/1567201811310050002 23410071

[B73] Mishra B. Patel B. B. Tiwari S. (2010). Colloidal nanocarriers: a review on formulation technology, types and applications toward targeted drug delivery. Nanomedicine 6, 9–24. 10.1016/j.nano.2009.04.008 19447208

[B74] Mokgokong R. Wang S. Taylor C. J. Barrand M. A. Hladky S. B. (2014). Ion transporters in brain endothelial cells that contribute to formation of brain interstitial fluid. Pflugers Arch. 466, 887–901. 10.1007/s00424-013-1342-9 24022703 PMC4006130

[B75] Mourtas S. Lazar A. N. Markoutsa E. Duyckaerts C. Antimisiaris S. G. (2014). Multifunctional nanoliposomes with curcumin-lipid derivative and brain targeting functionality with potential applications for alzheimer disease. Eur. J. Med. Chem. 80, 175–183. 10.1016/j.ejmech.2014.04.050 24780594

[B76] Nageeb El-Helaly S. Abd Elbary A. Kassem M. A. El-Nabarawi M. A. (2017). Electrosteric stealth rivastigmine loaded liposomes for brain targeting: preparation, characterization, *ex vivo*, bio-distribution and *in vivo* pharmacokinetic studies. Drug Deliv. 24, 692–700. 10.1080/10717544.2017.1309476 28415883 PMC8240971

[B77] Neves A. R. Lucio M. Martins S. Lima J. L. Reis S. (2013). Novel resveratrol nanodelivery systems based on lipid nanoparticles to enhance its oral bioavailability. Int. J. Nanomed. 8, 177–187. 10.2147/IJN.S37840 23326193 PMC3544347

[B78] Noble G. T. Stefanick J. F. Ashley J. D. Kiziltepe T. Bilgicer B. (2014). Ligand-targeted liposome design: challenges and fundamental considerations. Trends Biotechnol. 32, 32–45. 10.1016/j.tibtech.2013.09.007 24210498

[B79] Nunes S. Madureira A. R. Campos D. Sarmento B. Gomes A. M. Pintado M.. (2017). Solid lipid nanoparticles as oral delivery systems of phenolic compounds: overcoming pharmacokinetic limitations for nutraceutical applications. Crit. Rev. Food Sci. Nutr. 57, 1863–1873. 10.1080/10408398.2015.1031337 26192708

[B80] Organization W. H. (2006). Neurological Disorders: Public Health Challenges. Genève: World Health Organization (WHO/OMS).

[B81] Paliwal S. Tilak A. Sharma J. Dave V. Sharma S. Yadav R.. (2019). Flurbiprofen loaded ethosomes - transdermal delivery of anti-inflammatory effect in rat model. Lipids Health Dis. 18:133. 10.1186/s12944-019-1064-x 31170970 PMC6554971

[B82] Pandita D. Kumar S. Poonia N. Lather V. (2014). Solid lipid nanoparticles enhance oral bioavailability of resveratrol, a natural polyphenol. Food Res. Int. 62, 1165–1174. 10.1016/j.foodres.2014.05.059

[B83] Pardridge W. M. (2012). Drug transport across the blood-brain barrier. J. Cereb. Blood Flow Metab. 32, 1959–1972. 10.1038/jcbfm.2012.126 22929442 PMC3494002

[B84] Pardridge W. M. (2015). Targeted delivery of protein and gene medicines through the blood-brain barrier. Clin. Pharmacol. Ther. 97, 347–361. 10.1002/cpt.18 25669455

[B85] Pardridge W. M. Boado R. J. (2012). Reengineering biopharmaceuticals for targeted delivery across the blood-brain barrier. Meth. Enzymol. 503, 269–292. 10.1016/B978-0-12-396962-0.00011-2 22230573

[B86] Phachonpai W. Wattanathorn J. Muchimapura S. Tong-Un T. Preechagoon D. (2010). Neuroprotective effect of quercetin encapsulated liposomes: a novel therapeutic strategy against alzheimer's disease. Am. J. Appl. Sci. 7, 480–485. 10.3844/ajassp.2010.480.485

[B87] Poewe W. Antonini A. Zijlmans J. C. Burkhard P. R. Vingerhoets F. (2010). Levodopa in the treatment of parkinson's disease: an old drug still going strong. Clin. Interv. Aging 5, 229–238. 10.2147/CIA.S6456 20852670 PMC2938030

[B88] Pujara N. D. (2012). Self emulsifying drug delivery system: a novel approach. Int. J. Curr. Pharm. Res. 4, 18–23.

[B89] Pulgar V. M. (2018). Transcytosis to cross the blood brain barrier, new advancements and challenges. Front. Neurosci. 12:1019. 10.3389/fnins.2018.01019 30686985 PMC6337067

[B90] Re F. Cambianica I. Sesana S. Salvati E. Cagnotto A. Salmona M. (2011). Functionalization with apoe-derived peptides enhances the interaction with brain capillary endothelial cells of nanoliposomes binding amyloid-beta peptide. J. Biotechnol. 156, 341–346. 10.1016/j.jbiotec.2011.06.037 21763360

[B91] Rip J. Appeldoorn C. Manca F. Dorland R. Van Kregten J. Gaillard P. (2010). Receptor-Mediated Delivery of Drugs Across the Blood-Brain Barrier. Brussels: Pharmacology and Toxicology of the Blood-Brain Barrier: State of the Art, Needs for Future Research and Expected Benefits for the EU.

[B92] Rip J. Chen L. Hartman R. van den Heuvel A. Reijerkerk A. van Kregten J.. (2014). Glutathione pegylated liposomes: pharmacokinetics and delivery of cargo across the blood-brain barrier in rats. J. Drug Target 22, 460–467. 10.3109/1061186X.2014.888070 24524555 PMC4651142

[B93] Risau W. Wolburg H. (1990). Development of the blood-brain barrier. Trends Neurosci. 13, 174–178. 10.1016/0166-2236(90)90043-A 1693235

[B94] Ross C. A. Poirier M. A. (2004). Protein aggregation and neurodegenerative disease. Nat. Med. 10, S10–17. 10.1038/nm1066 15272267

[B95] Rotman M. Welling M. M. Bunschoten A. de Backer M. E. Rip J. Nabuurs R. J.. (2015). Enhanced glutathione pegylated liposomal brain delivery of an anti-amyloid single domain antibody fragment in a mouse model for alzheimer's disease. J. Control. Release 203, 40–50. 10.1016/j.jconrel.2015.02.012 25668771

[B96] Sadegh Malvajerd S. Azadi A. Izadi Z. Kurd M. Dara T. Dibaei M.. (2019). Brain delivery of curcumin using solid lipid nanoparticles and nanostructured lipid carriers: Preparation, optimization, and pharmacokinetic evaluation. ACS Chem. Neurosci. 10, 728–739. 10.1021/acschemneuro.8b00510 30335941

[B97] Sagare A. P. Deane R. Zlokovic B. V. (2012). Low-density lipoprotein receptor-related protein 1: a physiological abeta homeostatic mechanism with multiple therapeutic opportunities. Pharmacol. Ther. 136, 94–105. 10.1016/j.pharmthera.2012.07.008 22820095 PMC3432694

[B98] Salem H. F. Ahmed S. M. Hassaballah A. E. Omar M. M. (2015). Targeting brain cells with glutathione-modulated nanoliposomes: *in vitro* and *in vivo* study. Drug Des. Devel. Ther. 9, 3705–3727. 10.2147/DDDT.S85302 26229435 PMC4516201

[B99] Salvati E. Re F. Sesana S. Cambianica I. Sancini G. Masserini M.. (2013). Liposomes functionalized to overcome the blood-brain barrier and to target amyloid-beta peptide: the chemical design affects the permeability across an *in vitro* model. Int. J. Nanomed. 8, 1749–1758. 10.2147/IJN.S42783 23674890 PMC3652512

[B100] Sarkar S. Das N. (2006). Mannosylated liposomal flavonoid in combating age-related ischemia-reperfusion induced oxidative damage in rat brain. Mech. Ageing Dev. 127, 391–397. 10.1016/j.mad.2005.12.010 16480758

[B101] Scherrmann J.-M. (2002). Drug delivery to brain via the blood–brain barrier. Vascul. Pharmacol. 38, 349–354. 10.1016/S1537-1891(02)00202-1 12529929

[B102] Selkoe D. J. (2002). Alzheimer's disease is a synaptic failure. Science 298, 789–791. 10.1126/science.1074069 12399581

[B103] Sharma G. Lakkadwala S. Modgil A. Singh J. (2016). The role of cell-penetrating peptide and transferrin on enhanced delivery of drug to brain. Int. J. Mol. Sci. 17:806. 10.3390/ijms17060806 27231900 PMC4926340

[B104] Shi J. Wang Y. Luo G. (2012). Ligustrazine phosphate ethosomes for treatment of alzheimer's disease, *in vitro* and in animal model studies. AAPS PharmSciTech 13, 485–492. 10.1208/s12249-012-9767-6 22415639 PMC3364399

[B105] Smeyne M. Smeyne R. J. (2013). Glutathione metabolism and parkinson's disease. Free Radic. Biol. Med. 62, 13–25. 10.1016/j.freeradbiomed.2013.05.001 23665395 PMC3736736

[B106] Sokolik V. Berchenko O. Shulga S. (2017). Comparative analysis of nasal therapy with soluble and liposomal forms of curcumin on rats with alzheimer's disease model. J. Alzheimers. Dis. Parkinsonism 7:357 10.4172/2161-0460.1000357

[B107] Sokolik V. V. Koliada O. K. Shulga S. M. (2016). Effect of beta-amyloid peptide 42 on the dynamics of expression and formation of capital a, cyrillicbeta40, il−1beta, tnf alpha, il−6, il−10 by peripheral blood mononuclear cells in vitro and its correction by curcumin. Ukrain. Biochem. J. 88, 109–118. 10.15407/ubj88.01.109 29227593

[B108] Sokolik V. V. Shulga S. M. (2016). Effect of curcumin on accumulation in mononuclear cells and secretion in incubation medium of capital a, cyrillicbeta(40) and cytokines under local excess of capital a, cyrillicbeta(42)-homoaggregates. Ukrain. Biochem. J. 88, 83–91. 10.15407/ubj88.03.083 29235333

[B109] Spuch C. Navarro C. (2011). Liposomes for targeted delivery of active agents against neurodegenerative diseases (alzheimer's disease and parkinson's disease). J. Drug Deliv. 2011:469679. 10.1155/2011/469679 22203906 PMC3238398

[B110] Stefanis L. (2012). Alpha-synuclein in parkinson's disease. Cold Spring Harb. Perspect. Med. 2:a009399. 10.1101/cshperspect.a009399 22355802 PMC3281589

[B111] Tagalakis A. D. Kenny G. D. Bienemann A. S. McCarthy D. Munye M. M. Taylor H.. (2014). Pegylation improves the receptor-mediated transfection efficiency of peptide-targeted, self-assembling, anionic nanocomplexes. J. Control. Release 174, 177–187. 10.1016/j.jconrel.2013.11.014 24269968

[B112] Tellone E. Galtieri A. Russo A. Giardina B. Ficarra S. (2015). Resveratrol: a focus on several neurodegenerative diseases. Oxid. Med Cell Longev. 2015:392169. 10.1155/2015/392169 26180587 PMC4477222

[B113] Terdthai T.-U. Panakaporn W. Jintanaporn W. Wathita P. (2010). Cognitive-enhancing and antioxidant activities of quercetin liposomes in animal model of alzheimer's disease. Online J. Biol. Sci. 10, 84–91. 10.3844/ojbsci.2010.84.91

[B114] Tosi G. Fano R. A. Bondioli L. Badiali L. Benassi R. Rivasi F.. (2011). Investigation on mechanisms of glycopeptide nanoparticles for drug delivery across the blood-brain barrier. Nanomedicine 6, 423–436. 10.2217/nnm.11.11 21542682

[B115] Touitou E. Dayan N. Bergelson L. Godin B. Eliaz M. (2000). Ethosomes - novel vesicular carriers for enhanced delivery: characterization and skin penetration properties. J. Control. Release 65, 403–418. 10.1016/S0168-3659(99)00222-9 10699298

[B116] Uner M. Yener G. (2007). Importance of solid lipid nanoparticles (sln) in various administration routes and future perspectives. Int. J. Nanomed. 2, 289–300. 18019829 PMC2676658

[B117] Upadhyay R. K. (2014). Drug delivery systems, cns protection, and the blood brain barrier. BioMed Res. Int. 2014:869269 10.1155/2014/869269 25136634 PMC4127280

[B118] Vernier P. Moret F. Callier S. Snapyan M. Wersinger C. Sidhu A. (2004). The degeneration of dopamine neurons in parkinson's disease: Insights from embryology and evolution of the mesostriatocortical system. Ann. N Y Acad. Sci. 1035, 231–249. 10.1196/annals.1332.015 15681811

[B119] Wang D. G. Liu W. Y. Chen G. T. (2013). A simple method for the isolation and purification of resveratrol from polygonum cuspidatum. J. Pharm. Anal. 3, 241–247. 10.1016/j.jpha.2012.12.001 29403824 PMC5760951

[B120] Wang Y. Xia Z. Xu J. R. Wang Y. X. Hou L. N. Qiu Y.. (2012). Alpha-mangostin, a polyphenolic xanthone derivative from mangosteen, attenuates beta-amyloid oligomers-induced neurotoxicity by inhibiting amyloid aggregation. Neuropharmacology 62, 871–881. 10.1016/j.neuropharm.2011.09.016 21958557

[B121] Wang Y. Xu H. Fu Q. Ma R. Xiang J. (2011). Protective effect of resveratrol derived from polygonum cuspidatum and its liposomal form on nigral cells in parkinsonian rats. J. Neurol. Sci. 304, 29–34. 10.1016/j.jns.2011.02.025 21376343

[B122] Wang Z. Zhao Y. Jiang Y. Lv W. Wu L. Wang B.. (2015). Enhanced anti-ischemic stroke of zl006 by t7-conjugated pegylated liposomes drug delivery system. Sci. Rep 5:12651. 10.1038/srep12651 26219474 PMC4518266

[B123] Warren K. E. (2018). Beyond the blood: brain barrier: the importance of central nervous system (cns) pharmacokinetics for the treatment of cns tumors, including diffuse intrinsic pontine glioma. Front. Oncol. 8:239. 10.3389/fonc.2018.00239 30018882 PMC6037693

[B124] Woodle M. C. Papahadjopoulos D. (1989). Liposome preparation and size characterization. Meth. Enzymol. 171, 193–217. 10.1016/S0076-6879(89)71012-0 2593841

[B125] Wyss-Coray T. (2016). Ageing, neurodegeneration and brain rejuvenation. Nature 539, 180–186. 10.1038/nature20411 27830812 PMC5172605

[B126] Yang J. Song S. Li J. Liang T. (2014). Neuroprotective effect of curcumin on hippocampal injury in 6-ohda-induced parkinson's disease rat. Pathol. Res. Pract. 210, 357–362. 10.1016/j.prp.2014.02.005 24642369

[B127] Yang Z. Z. Zhang Y. Q. Wang Z. Z. Wu K. Lou J. N. Qi X. R. (2013). Enhanced brain distribution and pharmacodynamics of rivastigmine by liposomes following intranasal administration. Int. J. Pharm. 452, 344–354. 10.1016/j.ijpharm.2013.05.009 23680731

[B128] Yan-Hong L. Yong-Hua W. Yan L. Ling Y. (2006). Mdr1 gene polymorphisms and clinical relevance. Acta Genet. Sinica 33, 93–104. 10.1016/S0379-4172(06)60027-9 16529292

[B129] Zhao Z. Nelson A. R. Betsholtz C. Zlokovic B. V. (2015). Establishment and dysfunction of the blood-brain barrier. Cell 163, 1064–1078. 10.1016/j.cell.2015.10.067 26590417 PMC4655822

[B130] Zlokovic B. V. (2008). The blood-brain barrier in health and chronic neurodegenerative disorders. Neuron 57, 178–201. 10.1016/j.neuron.2008.01.003 18215617

